# Global incidence and prevalence of differentiated thyroid cancer in childhood: systematic review and meta-analysis

**DOI:** 10.3389/fendo.2023.1270518

**Published:** 2023-09-19

**Authors:** Mariacarla Moleti, Tommaso Aversa, Salvatore Crisafulli, Gianluca Trifirò, Domenico Corica, Giorgia Pepe, Laura Cannavò, Maria Di Mauro, Giuseppe Paola, Andrea Fontana, Fabrizio Calapai, Salvatore Cannavò, Malgorzata Wasniewska

**Affiliations:** ^1^ Department of Clinical and Experimental Medicine, University of Messina, Messina, Italy; ^2^ Department of Human Pathology of Adulthood and Childhood, University of Messina, Messina, Italy; ^3^ Department of Medicine, University of Verona, Verona, Italy; ^4^ Department of Diagnostics and Public Health, University of Verona, Verona, Italy; ^5^ Unit of Endocrinology, University Hospital Policlinico “G. Martino”, Messina, Italy; ^6^ Unit of Biostatistics, Fondazione IRCCS Casa Sollievo della Sofferenza, San Giovanni Rotondo, Italy; ^7^ Department of Chemical, Biological, Pharmaceutical and Environmental Sciences, University of Messina, Messina, Italy

**Keywords:** differentiated thyroid cancer, papillary thyroid carcinoma, follicular thyroid carcinoma, children, meta-analysis, incidence, prevalence

## Abstract

**Objective:**

Differentiated thyroid cancer (DTC) is rare in childhood and adolescence although it represents the most frequent endocrine malignancy in this population. DTC includes both papillary thyroid carcinoma (PTC) and follicular thyroid carcinoma (FTC). Most pediatric DTCs are PTCs, while FTCs are rare. To date, no systematic reviews on the global epidemiology of pediatric and adolescent DTC have been published. This systematic review and meta-analysis aims to estimate the overall incidence and prevalence of DTCs in patients aged 0–19 years.

**Methods:**

The systematic research was conducted from January 2000 to December 2021 through MEDLINE via PubMed, Cochrane Library, and Embase databases. Two separate meta-analyses were performed for PTC and FTC.

**Results:**

After the selection phase, a total of 15 studies (3,332 screened) met the inclusion criteria and are reported in the present systematic review. Five studies were conducted in Europe, five in North America, two in South America, one in Asia, one reported data for 49 countries and territories across the five continents, and one from both the USA and Africa. Most of the studies (*n* = 14) reported data obtained from national registries, and only one provided information collected from hospital medical records. Beyond the actual trend over time, our study reported a pooled global incidence rate (IR) of PTC and FTC in the pediatric age of 0.46 (95% CI: 0.33–0.59) and 0.07 (95% CI: 0.02–0.12) per 100,000 person-years, respectively. The highest IRs were recorded among Caucasian girls, and the lowest in black or other races/ethnicities.

**Conclusion:**

Our data confirm that DTC in the pediatric population is a rare condition. The pooled IRs of the studies included in this meta-analysis are ~0.5 for PTC, which is the most common histological type when both genders and all age groups are considered. The implementation of a prospective international registry on pediatric DTC, as part of the wider European Registries for Rare Endocrine Conditions, has been recently proposed. In addition to providing relevant information on the clinical behavior of this rare disease, standardization of data collection will be pivotal to fill current gaps and allow an accurate estimation of the real incidence and risk factors of DTC.

## 1 Introduction

Differentiated thyroid cancer (DTC) is rare in childhood and adolescence although it represents the most frequent endocrine malignancy in this population (1). DTC includes both papillary thyroid carcinoma (PTC) and follicular thyroid carcinoma (FTC), while poorly differentiated thyroid carcinoma (PDTC), anaplastic thyroid carcinoma (ATC), and medullary thyroid carcinoma (MTC) are subsets of thyroid cancer not included in this definition.

The Surveillance, Epidemiology, and End Results (SEER) database revealed that overall thyroid cancer accounts for 1.9% of all cancers in a population aged less than 20 years (2). The annual incidence of thyroid cancer in Americans aged 0–19 years has been estimated at 6.9 cases per million in the period 1975–2018 (2). However, the incidence rates (IRs) increase with age, and most cases are found in adolescents, with a predominance of female cases (3, 4). Specifically, the IRs increase from 0.43 in children aged 5–9 years to 3.5 in adolescents aged 10–14 years and up to 15.6 per million in those aged 15–19 years (5–7). While the rates are equally distributed between the male and female gender in prepubertal children, a clear female predominance in adolescence becomes evident, with the female-to-male ratio increasing up to 1:6 and making thyroid cancer the second most common malignancy in adolescent girls (6–8).

The increasing incidence over time is largely attributed to increased detection, though this does not account entirely for the rise in cases, and a true incidence increase cannot be definitely excluded (5). Jensen et al. recently reported an increase in the incidence of DTC presenting in young adults but not in children or adolescents in the Danish population (9).

Thyroid nodules are rarer in children than in adults but require prompt investigation, as the rate of malignancy is reported to be between 10% and 50%, significantly higher than in adults, in whom the reported rates range between 5% and 15% (10–12). Most cases of DTC in children and adolescents are PTC, while FTC is uncommon in this age group. Furthermore, DTC in children seems to differ in its behavior from that observed in adults, as children with DTC tend to present with more advanced disease (13–15), often with lymph node involvement at diagnosis (15, 16). Distant metastases, most commonly pulmonary, are also more frequent in children than in their adult counterpart (13, 14).

To date, no systematic reviews on the global epidemiology of pediatric and adolescent DTC have been published. Therefore, this systematic review and meta-analysis aimed to estimate DTC global incidence and prevalence in people aged 0–19 years, to evaluate the quality of study reporting, and to analyze the factors possibly contributing to trend modifications over time.

## 2 Material and methods

### 2.1 Literature search strategy and selection criteria

This systematic review and meta-analysis was conducted in accordance with The Preferred Reporting Items for Systematic Reviews and Meta-Analyses (PRISMA) statement (17) (**Supplementary Table 1**). A literature search on the epidemiology of pediatric DTC was carried out using the bibliographic databases MEDLINE and Embase from January 2000 to December 2021. Both databases were searched for terms related to DTC, prevalence, incidence, and epidemiology. Citations, titles, and abstracts were exported into Endnote X9. The complete search strategy for each database is provided in **Supplementary Table 2**.

Only original observational research articles written in English and reporting numerical and well-defined data on the epidemiology of pediatric DTC, including the number of cases, the underlying population, and the observation period, were considered. Narrative or systematic reviews and meta-analyses, as well as book chapters, editorials, and conference abstracts, were excluded; however, the references included in the narrative or systematic reviews and meta-analyses were screened to identify other potential studies to be included. Studies were also excluded if they used pharmacoeconomics or segregation analysis methods since, in the latter, epidemiological evaluations are based on mathematical models that make projections of the number of expected cases in a given population, thus concerning predicted and not actually observed incidence or prevalence (18). No geographic exclusion criteria were imposed.

After removing duplicates from the two different databases, six medically trained experts in pediatric endocrinology and pharmacoepidemiology (FC, LC, DC, MDM, GPe, GPa) screened individually the title and abstract of all records identified to remove articles that were clearly not relevant; the full texts of the articles were then independently reviewed by three experts (TA, MM, MW) to define whether they met the inclusion criteria or not. Any disagreements were resolved through discussion or, if consensus was not reached, through the intervention of a 10th expert (GT).

### 2.2 Data extraction and quality of study reporting assessment

Data from each included study were individually extracted by two authors (SCr, FC). The collected information included author(s) and year of publication, study catchment area, data source, prevalence/incidence type, study population, study period, study design, DTC definition, and the epidemiological estimate. For each included study, the incidence of the disease was defined as the number of new DTC cases per 100,000 person-years. The quality of study reporting was independently evaluated by two experts (SCr, FC) through a checklist specifically adapted for observational studies on the epidemiology of rare diseases from Strengthening the Reporting of Observational Studies in Epidemiology (STROBE) (19). An overall low, medium, and high score (see **Supplementary Document 1** for the complete quality assessment) was assigned to each included study, based on the following five fields: description of the study design and setting, eligibility criteria, study population, outcomes, and study participants. Disagreements in scoring were resolved through the intervention of a third expert (GT).

### 2.3 Statistical analyses

The meta-analysis of IRs was performed assuming that each study-specific rate was normally distributed and that the corresponding standard error (SE) was either provided by the authors or derived on the basis of the reported 95% confidence interval (CI) or *p*-value. A linear mixed effects model with random intercept was fitted to provide a pooled estimate of the IRs. The classical Cochran’s *Q* test and its derived inconsistency measure (*I* ^2^) were computed to assess the between-study heterogeneity, and it was claimed to be present when Cochran’s *Q* test *p*-value was <0.10 or *I* ^2^ >40% (19). Both study-specific and pooled epidemiological estimates were represented graphically, along with their 95% CI, on a forest plot. Two separate meta-analyses were performed for papillary and follicular thyroid carcinomas. Test for publication bias and metaregression were not performed due to the limited number of included studies (i.e., less than 10 studies) (20). Statistical analyses were performed using the R Foundation for Statistical Computing (version 4.2, package: *metafor*).

## 3 Results

### 3.1 Study selection and characteristics

**Figure 1:**
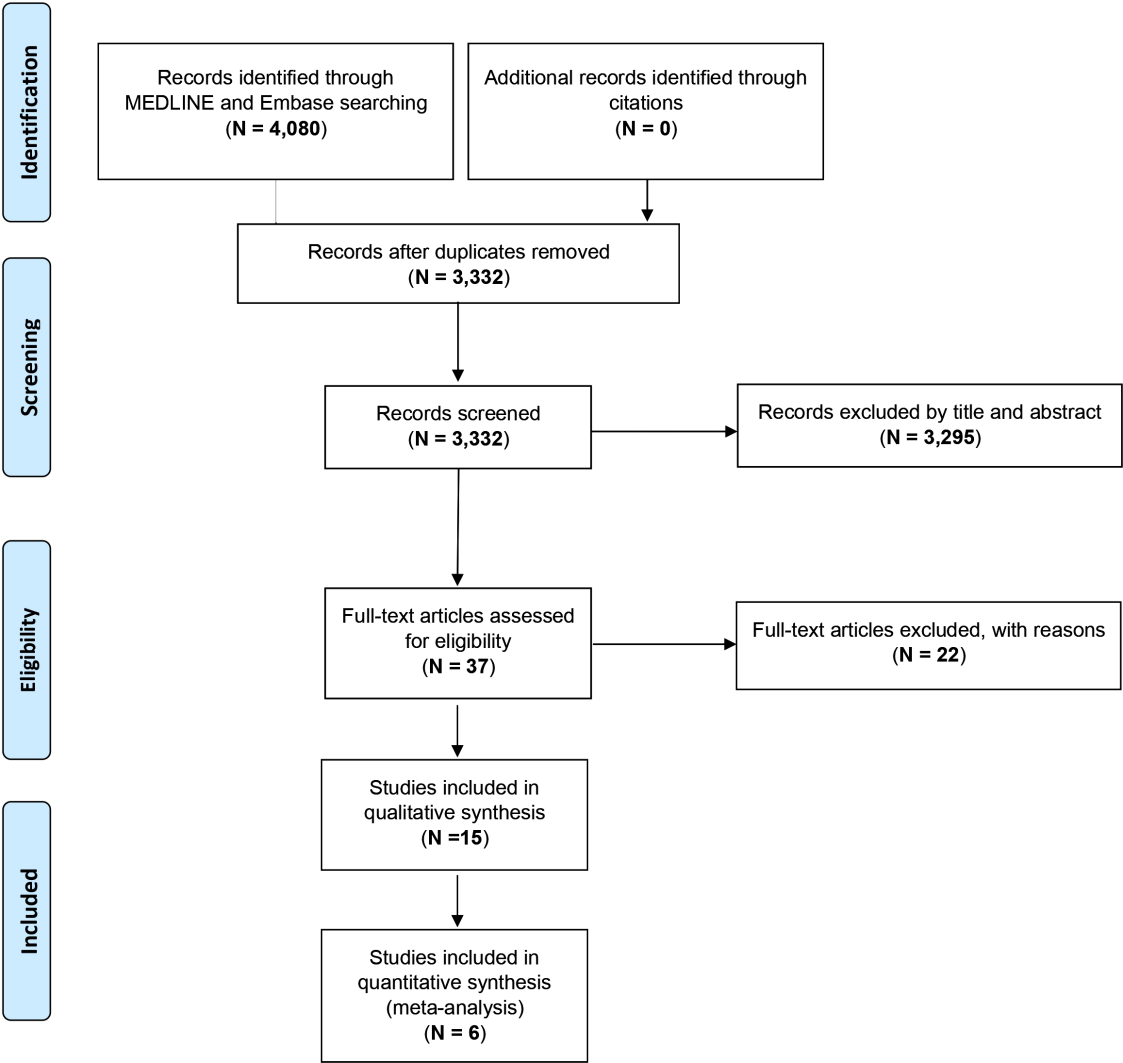
PRISMA flowchart showing the process of literature search and study selection.

The PRISMA flowchart describing the process of study selection is reported in **Figure 1**. A total of 4,080 studies were identified through the literature search. After the removal of duplicates (*n* = 748, 18.3%), 3,332 titles and abstracts were screened. Records excluded by title and abstract were 3,295, and only 37 (0.9%) full-text articles were retained for further evaluation. Among these, 15 (0.37%) studies met the inclusion criteria and were finally included in the systematic review (9, 21–34).

The characteristics of the included studies are shown in **Table 1**. Overall, five (35.7%) studies were conducted in Europe (9, 23, 27, 31, 32), five (35.7%) in North America (21, 22, 25, 26, 29), two (14.4%) in South America (24, 30), one (7.1%) in Asia (28), one (7.1%) reported data for 49 countries and territories across the five continents (33), and one from both the USA and Africa (34).

**Table 1:** Characteristics of the included studies investigating the epidemiology of differentiated thyroid cancer in childhood.

Ref.	First author (year)	Catchment area	Data source	Study period	Study population considered in the systematic review	Thyroid cancer definition	Epidemiological parameter	Included in the meta-analysis: yes or no (reason)
(21)	Aschebrook-Kilfoy (2013)	USA	National Cancer Institute’s Surveillance, Epidemiology, and End Results (SEER) program	1980–2009	PTC children aged 0–9 years and 10–19 years FTC children aged 0–9 years and 10–19 years	ICD-O-3 codes 8050, 8260, 8340-44, 8350, 8450, 8452, 8460 (PTC); ICD-O-3 codes 8290, 8330-32, 8335 (FTC)	Period incidence rates per 100,000 person-years	Yes
(22)	Bernier (2019)	USA	North American Association of Central Cancer Registries (NAACCR)	1998–2013	PTC children aged 0–9 years, 10–14 years, and 15–19 years FTC children aged 0–9 years, 10–14 years, and 15–19 years	ICD-O-3 codes 8050, 8052, 8130, 8260, 8340-44, 8450, 8452, 8460 (PTC); codes 8290, 8330-32, 8335 (FTC)	Age-standardized incidence rates per 1,000,000 person-years	Yes
(23)	Berontiene (2017)	Lithuania	Medical records from three hospitals in Lithuania (Hospital of the Lithuanian University of Health Sciences Kauno klinikos, Vilnius University Hospital Santariskiu Klinikos, Klaipedos University Hospital)	1980–2014	PTC children aged 7–9 years, 10–12 years, 13–15 years, and 16–18 years FTC children aged 7–9 years, 10–12 years, 13–15 years, and 16–18 years	Postoperative pathology examined at three main university hospitals in Lithuania	ASR per 1,000,000 person-years	No (epidemiological estimates of histological subtypes are not provided)
(24)	De Souza Reis (2020)	Brazil	Eleven population-based cancer registries encompassing five geographic regions of Brazil	2000–2013	PTC children aged 0–14 years FTC children aged 0–14 years	ICD-O-2 and ICD-O-3 codes 8050, 8052, 8130, 8260, 8340-44, 8450, 8452 (PTC); 8290, 8330-32, 8335 (FTC)	Period incidence per 1,000000	Yes
(25)	Golpanian (2015a)	USA	National Cancer Institute’s Surveillance, Epidemiology, and End Results (SEER) program	1973–2011	PTC children aged 0–4 years, 5–9 years, 10–14 years, and 15–19 years	ICD-O-3 all variants of PTC (codes not specified)	Period incidence per 100,000	Yes
(26)	Golpanian (2015b)	USA	National Cancer Institute’s Surveillance, Epidemiology, and End Results (SEER) program	1973–2011	FTC children aged 0–4 years, 5–9 years, 10–14 years, and 15–19 years	ICD-O-3 non-papillary types including follicular thyroid carcinoma, medullary thyroid carcinoma, and Hurtle cell carcinomas (codes not specified)	Period incidence per 100,000	No (in this series, FTCs were 54% of all non-papillary cancers and results only for FTC could not be excerpted)
(27)	Grønhøj (2018)	Denmark	Danish Cancer Registry	1978–2014	PTC children aged 0–4 years, 5–9 years, and 10–14 years FTC children aged 0–4 years, 5–9 years, and 10–14 years	ICD-10 papillary and follicular thyroid carcinoma, based on MORPHO-3 registration	Period incidence per 100,000	Yes
(28)	Lee (2021)	South Korea	National Health Information Database of the National Health Insurance Service	2004–2016	DTC children aged 0–9 years, 10–14 years, 15–17 years, and 18–19 years	ICD-10 code C73	ASR per 100,000 person-years	No (epidemiological estimates of histological subtypes are not provided)
(29)	Qian (2019)	USA	National Cancer Institute’s Surveillance, Epidemiology, and End Results (SEER) program	1973–2013	PTC children aged 0-9 years, 10–14 years, and 15–19 years FTC children aged 0–9 years, 10–14 years, and 15–19 years	ICD-O-3, C73, codes 8050, 8260, 8340-44, 8350, 8450-60 (PTC); 8290, 8330-35 (FTC)	Period incidence per 100,000	No (study period and data sources overlap with those used by Golpanian et al.)
(30)	Ramirez-Vick (2011)	Puerto Rico	Central Cancer Registry of Puerto Rico database	1985–2004	PTC children aged 0–19 years FTC children aged 0–19 years	ICD-O-3, codes 8050, 8052, 8130, 8260, 8340-44, 8450, 8452 (PTC); 8290, 8330-32, 8335 (FTC)	Incidence rate per 100,000 in 2004	No (epidemiological estimates of histological subtypes are not provided)
(31)	Russo (2017)	Italy	Sicilian Regional Register for Thyroid Cancer	2002–2009	PTC children aged <5 years, 5–9 years, 10–14 years, and 15–19 years	ICD 9, PTC (all variants) and FTC (all variants) (codes not specified)	Period incidence per 100,000	Yes
(9)	Schmidt Jensen (2018)	Denmark	Danish Cancer Registry; Danish Pathology data Bank; central population register	1980–2014	PTC children aged 0–17 years FTC children aged 0–17 years	ICD-O-3, MORPHO-3 codes 80503, 82603, 82903, 83303 (PTC); 83303, 83313, 83403 (FTC)	Annual Incidence per 100,000 in 2014	No (epidemiological estimates of histological subtypes are not provided)
(32)	Smailyte (2006)	Lithuania	Lithuanian Cancer Registry	1978–2003	PTC children aged 0–19 years FTC children aged 0–19 years	ICD-0-1 site codes 1930–1939 for the period 1978–1997 and ICD-O-2 site codes C730-739 for the period 1998–2003	Annual incidence per 100,000 in 2003	No (epidemiological estimates of histological subtypes are not provided)
(33)	Vaccarella (2021)	47 countries across five continents	159 registries from 47 countries and territories across the five continents	2008–2012	PTC children aged 0–9 years, 10–14 years, and 15–19 years FTC children aged 0–9 years, 10–14 years, and 15–19 years	ICD-O-3 codes for all variants of PTC and FTC (codes not specified)	ASR per 1,000,000 person-years	No (the 95% confidence intervals of the epidemiological estimates are not reported)
(34)	Woodruff (2010)	Nigeria–USA (Texas)	West African Center’s Cancer Registry Database Surgery database University of Texas Southwestern Medical Center at Dallas	1980–2004	PTC children aged 0–10 years and 11–20 years FTC children aged 0–10 years and 11–20 years	Diagnosis of DTC subtypes at both institutions based on WHO criteria (not otherwise specified)	Period prevalence in 1980–2004 in West Africa Period prevalence in 1997–2008 in UT Southwestern Medical Center	No (epidemiological estimates of histological subtypes are not provided)

Most of the studies (*n* = 14, 93.3%) reported data obtained from national registries (9, 21, 22, 24–34) and only one (23) provided information collected from hospital medical records.

Overall, incidence data were obtained over a median period of 25 years (range 4–40 years, IQR 14–35 years), and all the included studies stratified data by gender and, with few exceptions only (9, 24, 30, 32), according to two (21, 34) or ≥3 age groups (22, 23, 25–29, 31, 33).

Thyroid cancer definition was based on the International Classification of Diseases for Oncology (ICD-O) third edition (ICD-O-3) in just over half of the included studies (53.3%) [21, 22, 25, 26, 29, 30, 9, 34), on both the ICD-O-2 and ICD-O-3 in one study (24), on the ICD-O-1 and ICD-O-2 in one study (32), and according to the International Classification of Diseases (ICD) ninth (ICD-9) and 10th (ICD-10) edition in one (31) and two (27, 28) studies, respectively. The ICD edition used to define thyroid cancer was not specified in two studies (23, 34).

Six studies (42.8%) reported both tumor stage and size at diagnosis (22, 23, 25, 26, 29, 31), although in two of these studies (22, 29), information on the above characteristics was available only for a part of the study period. One additional study reported data on tumor stage only (32).

Of the 15 studies included in the systematic review, nine (60%) (9, 23, 26, 28–30, 32–34) were not included in the meta-analysis because no distinction between histological subtypes was applied in the calculation of the incidence rate or because neither the 95% CI nor the denominator used to calculate the prevalence and/or the incidence was reported in the full-text articles. Overall, the quality of study reporting was evaluated for 15 studies (**Supplementary Table 3**). In total, it was estimated as medium for 10 (66.7%) studies, as high for 3 (20.0%) studies, and as low for 2 (13.3%) studies.

### 3.2 Epidemiology of pediatric differentiated thyroid cancer

Fourteen studies (93.3%) reported the incidence of pediatric thyroid cancer, as either period/annual (9, 21, 23–27, 29–32) or age-standardized (22, 28) IRs, and only one study (34) estimated the period prevalence of pediatric thyroid cancer.

IRs for race/ethnicity were computed only in four of the included studies (4/15, 26.7%), all of which had been conducted in the USA and reported data from the SEER program (25, 26, 29) and the North American Association of Central Cancer Registries (NAACCR) (22). In all the above studies, the highest IRs were recorded among Caucasian girls, and the lowest in black or other races/ethnicities.

**Figure 2:**
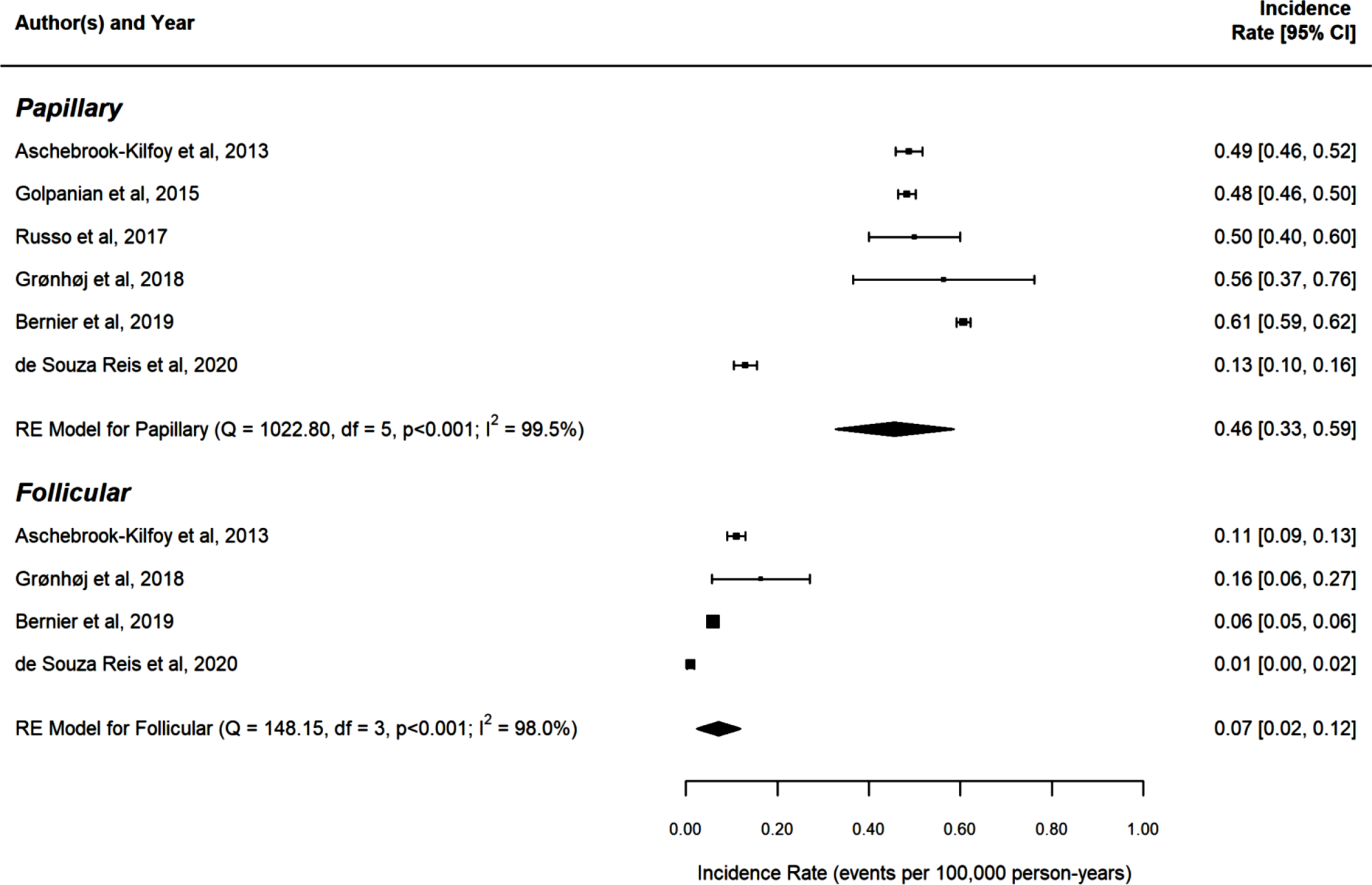
Forest plot of the thyroid carcinoma incidence rates, reported in each study (during its “study period”) per 100,000 person-years along with their 95% confidence intervals, stratified by thyroid carcinoma types (subgroups). Each square corresponds to the study-specific incidence rate (IR) estimate and its size is proportional to the inverse of the IR variance (ie the narrower the confidence interval, the targer the size of the square). The pooled estimate (centre line of diamond) and its confidence interval (lateral tips of diamond) are shown at the end of each subgroup. RE: Random-effects model (ie the pooled estimate was computed from a linear mixed-effects with a random intercept), Q: the value of Cochran's Q test, which follows a Chi-Square distribution with degrees of freedom (df) equal to the number of studies minus one p: p-value associated to the Q-test I2: the percentage of vanation across studies that is due to heterogeneity rather than chance and it is computed as follows: I2= P 100% x (Q-d)/Q.

Concerning DTC histotypes, 12 studies (80%) (9, 21–24, 27, 29–34) evaluated the IRs of both PTC and FTC separately, whereas one study focused on PTC only (25), one on FTC only (26), and one did not distinguish DTC subtypes (28). Among the studies included in the systematic review, IRs ranged from 0.13 (95% CI: 0.10–0.16) (24) to 0.61 (95% CI: 0.59–0.62) (22) cases per 100,000 persons for PTC and from 0.01 (95% CI: 0.00–0.02) (24) to 0.16 (95% CI: 0.06–0.27) (27) cases per 100,000 persons for FTC. The pooled IRs of the studies included in the meta-analysis were 0.46 [95% CI: 0.33–0.59] for PTC and 0.07 [95% CI: 0.02–0.12] for FTC when both sexes and all age groups were considered (**Figure 2**). Considerable heterogeneity was detected among the studies exploring both PTC (*Q* = 1,022.80, *df* = 5, *p* < 0.001; *I* ^2 = ^99.5%) and FTC (*Q* = 148.15, *df* = 3, *p* < 0.001; *I* ^2 = ^98%) IRs. Due to the limited number of included studies (<10), both metaregression and test for publication bias could not be performed.

## 4 Discussion

The prevalence of thyroid nodules in childhood and adolescence has been estimated to range between 0.5% and 2%, according to the method used for their screening (i.e., ultrasonography or palpation), the considered nodular size (i.e., only those having dimensions of more than 10 mm or even smaller nodules), and the background nutritional iodine status (35–39). While most of the thyroid nodules detected in this age group are benign, the risk of a nodule >10 mm being malignant is approximately two- to threefold higher in children than in adults (20%–25% vs. 5%–10%) (10, 11). Also, epidemiological data indicate that the worldwide incidence of DTC in pediatric patients is rising, this trend substantially mirroring the one reported in the adult population (21–25, 28, 29, 32, 33, 40, 41). In this regard, a recent large population-based registry study focused on the global patterns and trends in the incidence of thyroid cancer in children and adolescents analyzing data from 49 countries and territories worldwide (33). Despite the considerable variability in adjusted standardized ratios (ASRs) observed between countries, a clear temporal increase in incidence rates across geographically and ethnically divergent populations was evident, particularly among girls in the 10–19-year age group (33). Whether this trend (similar to what is assumed for the adult population) may be accounted for by overdetection of subclinical lesions is plausible, it is currently unproven. As a matter of fact, a US study reported a significant increase between 1998 and 2013 in the rates of either small (<1 cm) or large (>2 cm) tumors, as well as in diseases at early (DTC confined to the thyroid gland) or late (DTC with distant metastases) stages, with the above trends over the time period being significant for the age group 10–19 years and across racial and ethnic groups (22). Noteworthy, the temporal distribution of the recorded cases was skewed toward the last years, with approximately half of the newly diagnosed cases being detected in the last 6 years of the 16-year study period. In our view, rather than driven by significant changes in environmental/behavioral factors potentially affecting thyroid cancer incidence, this trend may argue in favor of increased medical surveillance, likely related to the overall growing concern toward DTC. Emblematic in this regard are the findings of a thyroid ultrasonography mass screening program implemented in Fukushima following the 2011 Nuclear Power Plant Accident and aimed at establishing the baseline prevalence of thyroid cancer among children and adolescents living in areas with different degrees of radiation exposure (42). An overall, and unexpected, prevalence of 37.3 per 100,000 childhood thyroid cancer was recorded in this study, but no significant differences were found between areas with the highest radioactive contamination and those with minimal radiation exposure. According to the authors, since recruitment in this study was carried out within the putative latent period for radiation-related cancers, the larger-than-expected number of cancers detected among children and adolescents is likely consistent with an overdiagnosis due to mass screening rather than an actual increase in DTC occurrence (42).

More recently, an observational study compared data collected by the International Agency for Research on Cancer (IARC) through the “Cancer Incidence in 5 continents plus” (CI5 plus) project with those of the latest report from the Global Cancer Observatory (GLOBOCAN 2020 project). An overall increasing prevalence of thyroid cancer was found in the age groups 10–19, with female late adolescents presenting a much higher incidence with respect to girls aged 10–14 years old (43).

Beyond the actual trend over time, our study reported a pooled global IR of PTC and FTC in the pediatric age of 0.46 (95% CI: 0.33–0.59] and 0.07 (95% CI: 0.02–0.12) per 100,000 person-years. When IRs for PTC and FTC were computed separately, the IR for PTC was almost 7 times higher than for FTC [0.46 (95% CI: 0.33–0.59) vs. 0.07 (95% CI: 0.02–0.12)], thus confirming the papillary histotype to be the more prevalent also in the pediatric population. It is worth noting that these estimates do not include DTC occurring in children who had been (potentially) exposed to radioactive fallout from nuclear accidents (i.e., Chernobyl or Fukushima power plant accidents), since radiation exposure represents a known risk factor for thyroid cancer and, thus, a clear confounding factor.

Unfortunately, we were not able to obtain more detailed estimates of thyroid cancer incidence according to gender and age groups due to several reasons. First, most of the studies included in the meta-analysis did not report detailed rates by sex for a precise estimate to be made. Second, the age groups examined were highly heterogeneous, thus precluding the possibility of exploring differences within the category of children/adolescents, between younger and older subjects, or between prepubertal children and adolescents. The above issues deserve special attention because of the potential influence of pubertal changes on thyroid cancer incidence in both boys and girls. In this regard, Zhao et al. (41) retrospectively examined cases of thyroid cancer from 2004 to 2017 in patients aged <10 (prepubertal), 10–15 (pubertal) and >15 (postpubertal) years and found that the annual proportion of total cases increased from 3% to 8% for <10-year-old children, from 31% to 40% for 10–15-year-old children, and from 52% to 66% for >15-year-old children (41). It could be argued that age groups as a proxy measure for defining puberty may not reliably reflect the pubertal status of all subjects, especially because of ethnic and environmental disparities in pubertal development (44). Nonetheless, the risk of DTC dramatically increases among postpubertal female and male subjects, and therefore, considering as a *unicum* an age group including either children or subjects with fully completed pubertal development may result in incorrect estimates.

Possible explanations for the observed increased risk of DTC in postpubertal female subjects compared with their male counterparts include mechanisms involving female sex hormones, with estrogen and estrogen receptors (ERs) being the most investigated target of current research. Several *in-vitro and* animal studies have evaluated the influence of sex steroids on the proliferation of thyroid cells, although considerable discrepancies with respect to ER expression patterns in thyroid cancer tissues actually exist (43, 45, 46).

Another potential limitation to a comprehensive data analysis comes from the lack of information on the nutritional iodine status of the populations under examination, with only one study comparing the patterns of DTC with respect to changes in iodine supply at the population level (34). This study showed a clear shift at any age toward a preponderance of PTC over FTC occurring in parallel with iodine nutrition improvement, although the rates of FTC still exceeded those reported in countries with well-established iodine sufficiency (34). These data confirm previous findings in adults showing a higher percentage of the more aggressive FTC in iodine-deficient areas (47) and the clear temporal relationship in many countries between the implementation of iodine prophylaxis programs and a relative increase in the incidence of PTC (48). Even more importantly, iodine deficiency increases the thyroid uptake of radioactive iodine and the proliferation rate of thyroid cells (49). Both the above effects facilitate the occurrence of thyroid cancer after radiation exposure, especially in children because of the highest sensitivity to radiation in this age category (50, 51). Paradigmatic in this regard is the marked increase in the incidence of thyroid cancer as early as 5 years following the accident at the nuclear power plant at Chernobyl in Ukraine—an iodine-deficient region—in children who were aged 0–5 years at the time of the accident and for whom no timely iodine prophylaxis measures were adopted (49).

The high sensitivity of the thyroid gland to the carcinogenic effects of ionizing radiation during childhood and adolescence is also evidenced by the radiation-related excess risk of second primary thyroid cancer after radiotherapy to the cervical region for a childhood cancer (52–54). Presently available evidence indicates that after a mean dose to the thyroid as low as 0.05 Gy to 0.1 Gy during childhood, a significant thyroid cancer risk is evident within 5 to 10 years of childhood exposure to radiation and remains elevated for potentially many decades (53). Also, the risk of developing a second primary DTC has been reported to be remarkably increased in children receiving alkylating agents in combination with radiation doses up to 20 Gy (53), as well as among survivors of pediatric hematopoietic stem cell transplantation conditioned with chemotherapy alone (55). Of the studies included in our systematic review, only three (9, 21, 22) specifically excluded second primary thyroid tumors from analysis, the remaining either not reporting this information (24–27, 29–32, 34) or admittedly including second primary thyroid tumors (23, 28). A recently published Italian registry-based study showed that thyroid cancer as a second primary tumor was diagnosed more frequently than in the general population, the overall standardized IR being 1.49 (95% CI: 1.42–1.55) but as high as 6.1 (95% CI: 2.9–11.2) or 4.4 (95% CI: 2.2–7.8) after acute lymphoid leukemia and bone cancers, respectively (56). Unfortunately, no data specifically addressing the pediatric population are reported in this large study, which would have been helpful to understand the actual impact of previous cancers (and related therapy) on thyroid cancer occurrence/diagnosis in this age group.

The high between-study heterogeneity should also be acknowledged. This could be partly explained by the characteristics of the total reference population in each included study or data collection methods (e.g., claims databases, electronic medical records, or disease registries).

In conclusion, our data indicate that DTC in the pediatric population is a rare condition, the pooled IRs of the studies included in this meta-analysis being as low as ~0.5 for PTC, which is by far the most common histotype when both sexes and all age groups are considered. The implementation of a prospective international registry on pediatric DTC, as part of the wider European Registries for Rare Endocrine Conditions, has been very recently proposed (57). In addition to providing relevant information on the clinical behavior of this rare disease, which can be helpful in offering patients evermore tailored therapeutic strategies (58), standardization of data collection will be pivotal to fill current gaps and allow an accurate estimation of the real incidence and risk factors of DTC.

## Author contributions

MM: Conceptualization, Data curation, Investigation, Validation, Writing – original draft, Writing – review & editing. TA: Data curation, Investigation, Writing – original draft, Writing – review & editing, Validation. SCr: Investigation, Methodology, Validation, Writing – original draft, Writing – review & editing. GT: Conceptualization, Supervision, Validation, Writing – review & editing. DC: Data curation, Investigation, Validation, Writing – review & editing. GPe: Data curation, Investigation, Validation, Writing – review & editing. LC: Data curation, Investigation, Validation, Writing – review & editing. MDM: Data curation, Investigation, Validation, Writing – review & editing. GPa: Data curation, Investigation, Validation, Writing – review & editing. AF: Methodology, Validation, Writing – review & editing. FC: Data curation, Investigation, Methodology, Validation, Writing – review & editing. SCa: Conceptualization, Supervision, Validation, Writing – review & editing. MW: Conceptualization, Data curation, Supervision, Writing – original draft, Writing – review & editing, Validation.
