# Supplementary material for: Global incidence and prevalence of differentiated thyroid cancer in childhood: systematic review and meta-analysis
Source: Front Endocrinol (Lausanne). 2023 Sep 19;14:1270518. doi: 10.3389/fendo.2023.1270518 (PMC10546309; doi:10.3389/fendo.2023.1270518)
Supplement: Supplementary file 3 [file Table_2.docx]

**Supplementary Table 2.** Literature search strategies

| **Embase** | | |
| --- | --- | --- |
| **Search** | **Query** | **Items found** |
| #19 | #16 AND (2000:py OR 2001:py OR 2002:py OR 2003:py OR 2004:py OR 2005:py OR 2006:py OR 2007:py OR 2008:py OR 2009:py OR 2010:py OR 2011:py OR 2012py OR 2013:py OR 2014:py OR 2015:py OR 2016:py OR 2017:py OR 2018:py OR 2019:py OR 2020:py OR 2021:py) AND ([adolescent/lim OR [child]/Iim OR [embryo]/lim OR [fetus]/lim OR [infant]/lim OR [newborn]/lim OR [preschool]/Iim OR [school]/lim OR [young adult]/lim) AND ('case control study'/de OR 'case report'/de OR 'case study'/de OR 'clinical article'/de OR 'clinical study/de OR 'cohort analysis'/de OR 'comparative study'/de OR 'controlled study'/de OR 'cross sectional study/de OR 'evidence based practice'/de OR 'family study'/de OR 'human'/de OR 'human tissue/de OR 'intermethod comparison'/de OR 'Interview'/de OR 'longitudinal study'/de OR 'major clinical study'/de OR 'medical record review'/de OR 'meta analysis'/de OR 'model'/de OR 'multicenter study/de OR 'normal human'/de OR 'observational study'/de OR 'outcomes research'/de OR 'pilot study'/de OR 'population based case control study'/de OR 'proportional hazards model'/de OR 'prospective study'/de OR 'questionnaire'/de OR 'retrospective study'/de OR 'statistical model'/de OR 'study design'/de OR 'systematic review'/de OR 'trend study'/de) | 2,980 |
| #18 | #16 AND (2000:py OR 2001:py OR 2002:py OR 2003:py OR 2004:py OR 2005:py OR 2006:py OR 2007:py OR 2008:py OR 2009:py OR 2010:py OR 2011:py OR 2012py OR 2013:py OR 2014:py OR 2015:py OR 2016:py OR 2017:py OR 2018:py OR 2019:py OR 2020:py OR 2021:py) AND ([adolescent/lim OR [child]/Iim OR [embryo]/lim OR [fetus]/lim OR [infant]/lim OR [newborn]/lim OR [preschool]/Iim OR [school]/lim OR [young adult]/lim) | 3,016 |
| #17 | #16 AND (2000:py OR 2001:py OR 2002:py OR 2003:py OR 2004:py OR 2005:py OR 2006:py OR 2007:py OR 2008:py OR 2009:py OR 2010:py OR 2011:py OR 2012py OR 2013:py OR 2014:py OR 2015:py OR 2016:py OR 2017:py OR 2018:py OR 2019:py OR 2020:py OR 2021:py) | 4,290 |
| #16 | #11 AND #15 | 4,934 |
| #15 | #12 OR #13 OR #14 | 2,292,312 |
| #14 | prevalence OR incidence:ti,ab | 2,151,622 |
| #13 | 'prevalence'/exp | 802,271 |
| #12 | 'incidence’/exp | 529,072 |
| #11 | #6 AND #10 | 21,941 |
| #10 | #7 OR #8 OR #9 | 3,965,145 |
| #9 | ('preschool'/exp OR preschool) AND ('child’/exp OR child) | 653,367 |
| #8 | ('minors'/exp OR minors OR 'boy'/exp OR boy OR boys OR boyhood OR girl* OR infant* OR kid OR kids OR 'chlid'/exp OR child OR children* OR childhood* OR childcare* OR 'schoolchild’/exp OR schoolchild OR adolescen* OR juvenil* OR youth* OR teen* OR preteen* OR underage* OR under) AND age:ti,ab | 1,394,227 |
| #7 | 'child'/exp OR 'child' | 3,438,886 |
| #6 | #1 OR #2 OR #3 OR #4 OR #5 | 370,829 |
| #5 | (thyroid NEAR/3 malignancy):ti,ab | 3,055 |
| #4 | 'adenocarcinoma'/exp | 246,545 |
| #3 | thyroid NEAR/3 (neoplasm OR tumor) | 22,789 |
| #2 | (‘thyroid’exp OR thyroid) AND ('neoplasm'/exp OR neoplasm) | 130,070 |
| #1 | thyroid AND neoplasm | 22,803 |
| **Medline** | | |
| **Search** | **Query** | **Items found** |
| #1 | Thyroid Neoplasms/ | 50,701 |
| #2 | exp Thyroid Neoplasms/ | 54,082 |
| #3 | (thyroid adj3 (neoplasm or tumor)).tw. | 3,590 |
| #4 | Adenocarcinoma, Follicular/ | 3,948 |
| #5 | (thyroid adj3 malignancy).ti,ab. | 2,069 |
| #6 | #1 or #2 or 3 or 4 or 5 | 55,752 |
| #7 | exp Child/ | 1,952,806 |
| #8 | (minors or boy or boys or boyhood or girl* or infant* or kid or kids or child or children* or childhood* or childcare* or schoolchild* or adolescen* or juvenil* or youth* or teen* or preteen* or underage* or under age*).ab,ti. | 2,076,865 |
| #9 | child, preschool/ or preschool.mp. | 943,060 |
| #10 | 7 or 8 or 9 | 296,916,7 |
| #11 | 6 and 10 | 6,233 |
| #12 | exp Prevalence/ | 304,509 |
| #13 | exp Incidence/ | 272,612 |
| #14 | Thyroid Neoplasms/ep [Epidemiology] | 3,718 |
| #15 | (prevalence or incidence).ti,ab. | 1,375,737 |
| #16 | 12 or 13 or 14 or 15 | 1,551,479 |
| #17 | 11 and 16 | 1,762 |
| #18 | 11 and 16 (period: 2000-2021) | 1,100 |

|  |  |
| --- | --- |
|  |  |
|  |  |
|  |  |
